# Immunization With Skp Delivered on Outer Membrane Vesicles Protects Mice Against Enterotoxigenic *Escherichia coli* Challenge

**DOI:** 10.3389/fcimb.2018.00132

**Published:** 2018-05-01

**Authors:** Michael P. Hays, Diane Houben, Yang Yang, Joen Luirink, Philip R. Hardwidge

**Affiliations:** ^1^Diagnostic Medicine/Pathobiology, College of Veterinary Medicine, Kansas State University, Manhattan, KS, United States; ^2^Section Molecular Microbiology, Department of Molecular Cell Biology, Faculty of Earth and Life Sciences, VU University, Amsterdam, Netherlands; ^3^Abera Bioscience AB, Stockholm, Sweden

**Keywords:** enterotoxigenic *Escherichia coli*, vaccines, outer membrane vesicles, mouse models, infection

## Abstract

Outer membrane vesicles (OMVs) are promising vaccine components because they combine antigen and adjuvant in a single formulation. Detoxified *Salmonella enterica* strains that express penta-acylated lipid A retain OMV immunogenicity but with reduced reactogenicity. We have previously shown that a recombinant form of the enterotoxigenic *Escherichia coli* (ETEC) 17 kilodalton protein (Skp) protects mice in a pulmonary challenge model, when fused to the glutathione-S-transferase (GST) epitope and combined with cholera toxin. Here we compared directly the efficacy of expressing Skp in detoxified *Salmonella* OMVs to GST-Skp for their ability to protect mice against ETEC challenge. We observed that the display of Skp on OMVs, in the absence of exogenous adjuvant, protects the mice as well as the recombinant GST-Skp with adjuvant, showing that we can achieve protection when antigen and adjuvant are administered as a single formulation. Collectively, these data demonstrate the utility of using OMVs for the expression and display of antigens for use in vaccine development and validate previously published work demonstrating that immunization with Skp is efficacious in protecting mice against ETEC challenge.

## Introduction

Enterotoxigenic *Escherichia coli* (ETEC) strains are important bacterial pathogens of both humans and livestock (Fleckenstein et al., 2010). These organisms cause diarrhea by colonizing the small intestine and producing heat-labile (LT) and/or heat-stable (ST) enterotoxins that induce water and electrolyte loss from the intestines (Nataro and Kaper, 1998). The lack of a successful, licensed ETEC vaccine is attributed to a number of general limiting factors and challenges that hamper the development of vaccine candidates and their downstream implementation. More specific to ETEC is the issue of heterogeneity of colonization factors (CFs) among ETEC strains. At least 25 immunologically distinct CFs produced by ETEC strains that cause diarrhea in humans have been identified. Most current technology is equipped to incorporate only one colonization factor. The variability among strains of relevance to human health makes the development of vaccines with sufficient coverage of ETEC strains extremely challenging and increases the likelihood that vaccine candidates will fail at later stages of development.

We previously characterized the efficacy of using the ETEC Skp protein as a vaccine candidate and found that a recombinant fusion of Skp to the glutathione-S-transferase (GST) epitope was efficacious in protecting mice when this recombinant protein was used as an immunogen in a pulmonary challenge model (Kumar et al., 2015; Hays et al., 2016). Vaccination with Skp-GST protected mice from an otherwise lethal dose of ETEC, reduced ETEC burdens in the lungs, and enhanced IgA responses (Kumar et al., 2015; Hays et al., 2016). These experiments were conducted using cholera toxin (CT) as an adjuvant (Kumar et al., 2015; Hays et al., 2016).

Outer membrane vesicles (OMVs) are spherical nanostructures of 30–200 nm diameter that are ubiquitously released from the outer membrane (OM) of Gram-negative bacteria. OMVs are promising vaccine candidates because they combine antigen and adjuvant in a single formulation (Ellis and Kuehn, 2010; Underhill and Goodridge, 2012; Gerritzen et al., 2017). OMV-based vaccines are already safely and successfully in use in humans (Esposito et al., 2014). The use of OMVs has gained interest since heterologous proteins were successfully integrated into vesicles (Kesty and Kuehn, 2004; Kim et al., 2008; Schild et al., 2009; Schroeder and Aebischer, 2009; Muralinath et al., 2011). *Vibrio cholerae*-derived OMVs have successfully been used to induce an antibody-mediated immune response in mice (Muralinath et al., 2011). *Salmonella* OMVs that contain the pneumococcal protein PspA in their lumen can induce protective immune responses when used as vaccine against *S. pneumoniae* in mice (Kim et al., 2008). OMVs can be used as a booster vaccine to increase the antigen-specific antibody responses in mice (Schild et al., 2009).

Accumulating evidence shows that both the magnitude and the range of the immune response can be improved by displaying antigens at the surface of OMVs (Alaniz et al., 2007). Therefore, the *E. coli* autotransporter Hemoglobin protease (Hbp) was developed into a platform for simultaneous surface display of multiple heterologous antigens, using a side-domain replacement strategy (Jong et al., 2012). The heterologous antigens are fused to the stable ~100 Å α-helical core structure of Hbp, resulting in optimal exposure of the proteins at a distance from the cell surface.

The Hbp with heterologous antigens are expressed on a detoxified, attenuated, and hypervesiculating *Salmonella* Typhimurium strain. For detoxification, the *msbB* gene (an acyl transferase of lipid A, the endotoxin component of LPS) was deleted. This deletion results in penta-acylated lipid A, which is less reactogenic compared to wild type LPS, but retains its immunogenicity (Kong et al., 2011; Kuipers et al., 2017). OMVs are shed in high amounts from this strain due to a compromised Tol-Pal system and show efficient surface exposure of the Hbp-antigen fusions, thus providing a safe, non-living vaccine platform for antigen display (Jong et al., 2014).

Here we quantified the extent to which presentation of ETEC Skp on detoxified OMVs would be sufficient to protect mice against ETEC challenge in the absence of an exogenous adjuvant.

## Materials and methods

### Ethics statement

The Kansas State University Institutional Animal Care and Use Committee approved the animal procedures (IACUC protocol #3900) in the context of the Kansas State University Animal Welfare Assurance Number A3609-01, in compliance with the Public Health Service (PHS) Policy on Humane Care and Use of Laboratory Animals. The experiments were also approved by the Kansas State University Institutional Biosafety Committee.

### OMV cloning and preparation

The HbpD(Δd1) expression plasmid has a pEH3 backbone (Hashemzadeh-Bonehi et al., 1998) and has been described previously (Jong et al., 2012). N-terminal (Fragment 1) and C-terminal (Fragment 2) Skp fragments were amplified with flanking SacI/BamHI sites, using the ETEC H10407 *skp* gene as a template with forward primers skpF1-InFu-Fw (5′-ggaagtcttgcggggagctccGCTGACAAAATTGCAATCGTC-3′), SkpF1-InFu-Fw (5′-taccgctgccggatccTTCCAGCTTAGTGCGATCGC-3′), SkpF2-InFu-Fw (5′-ggaagtcttgcggggagctccCAGGCTAAAATGAAAAAGCTGC-3′), and reverse primer SkpF2-InFu-Rv (5′-taccgctgccggatccTTTAACCTGTTTCAGTACGTCG-3′). The resulting PCR amplicons were digested with SacI and BamHI and inserted into the *hbp orfs* of plasmids pHbpD(Δd1) or pHbpD(Δd2) (Jong et al., 2012), which had been digested with the same restriction enzymes. This resulted in plasmids pHbpD(Δd1)-SkpF2 and pHbpD(Δd2)-SkpF1. To create a SkpF1F2 combination construct, the XbaI/NdeI fragment of pEH3-HbpD(Δd2)-SkpF1 was replaced by the XbaI/NdeI fragment of pEH3-HbpD(Δd1)-SkpF2, resulting in pEH3-HbpD-SkpF1F2.

The OMV production strain SL3261Δ*tolRA*Δ*msbB* (Kuipers et al., 2017) carrying expression plasmid pHbpD(Δd1) or pEH3-HbpD-SkpF1F2 was grown at 30°C in LB medium lacking NaCl (LB-0), supplemented with 2 mM CaCl_2_, 2 mM MgCl_2_ and 0.2 % glucose. Chloramphenicol (30 μg/ml) and Kanamycin (25 μg/ml) were added, when appropriate.

SL3261Δ*tolRA*Δ*msbB* cells harboring expression plasmids were grown overnight, after which they were subcultured in fresh medium to an OD_660_ of 0.07 and grown for 7 h until they reached an OD_660_ of 1.0. Cells were again subcultured in fresh medium to an OD_660_ of 0.02 and growth was continued overnight in the presence of 50 μM Isopropyl β-D-1-thiogalactopyranoside (IPTG) to induce protein expression. The next morning, OMVs were isolated from the culture supernatants as described previously (Kuipers et al., 2015). OMVs were finally resuspended in PBS containing 15% glycerol (1 OD unit of OMVs per μl). An amount of 1 OD unit of OMVs is derived from 1 OD_660_ unit of cells. Protein profiles of OMV samples were analyzed using SDS-PAGE and Coomassie G-250 (BioRad) staining. Densitometric analysis on Coomassie-stained gels was carried out using a Molecular Imager GS-800 Calibrated Densitometer and Quantity One software (Biorad). Proteinase K accessibility of OMV proteins was analyzed as described (Daleke-Schermerhorn et al., 2014). Skp-GST was purified as described previously (Kumar et al., 2015).

### Mouse infections

Female BALB/c mice were obtained from the Jackson Laboratory (Bar Harbor, Maine), housed in microisolator cages, and provided with food and water *ad libitum*. For vaccination studies, purified OMVs were administered at a concentration of eight optical density 660 nm (OD660) equivalents per 5 μl. OMVs (5 μl) were administered intranasally to the external nares of mice that were lightly anesthetized with isoflurane. Two identical booster doses were administered 2- and 4-weeks subsequent to the initial vaccination. Skp-GST was administered intranasally at 20 μg/dose with 2.5 μg of cholera toxin (Sigma-Aldrich) in 25 μl PBS. Booster doses were administered 2- and 4-weeks after the initial vaccination.

After the 6-week immunization regimens, mice were challenged intranasally with 10^8^ CFUs ETEC H10407. This strain was cultivated on CFA agar at 37°C for 18 h. ETEC was scraped off CFA agar and then suspended and diluted in sterile PBS. To quantify changes to mouse clinical signs of illness as a function of ETEC challenge, we observed mice every 8 h after challenge until the study is terminated at up to 7 days post-challenge. We recorded the clinical signs of illness as a function of time. Mice were euthanized and necropsied if one or more clinical signs of illness (lack of responsiveness to stimulation, hunched posture, ruffled hair coat, dehydration) were observed. Lungs were homogenized, serially diluted in PBS, and plated on MacConkey agar to enumerate ETEC. Fecal pellets were collected for quantification of IgA as described previously (Hays et al., 2016).

### Statistical analyses

Differences in mouse survival as a function of time after ETEC challenge were analyzed using Log-rank tests. Differences in both ETEC loads in mouse lungs and in fecal IgA concentrations were analyzed using Kruskal-Wallis tests. Asterisks indicate significant differences at *p* < 0.05.

## Results and discussion

We previously reported the efficacy of immunizing mice against Skp in subsequent protection against ETEC H10407, using CT as an adjuvant. Here we tested the hypothesis that OMV-mediated delivery of Hbp-Skp fusions would protect mice against ETEC challenge in the absence of an exogenous adjuvant.

**Figure 1:**
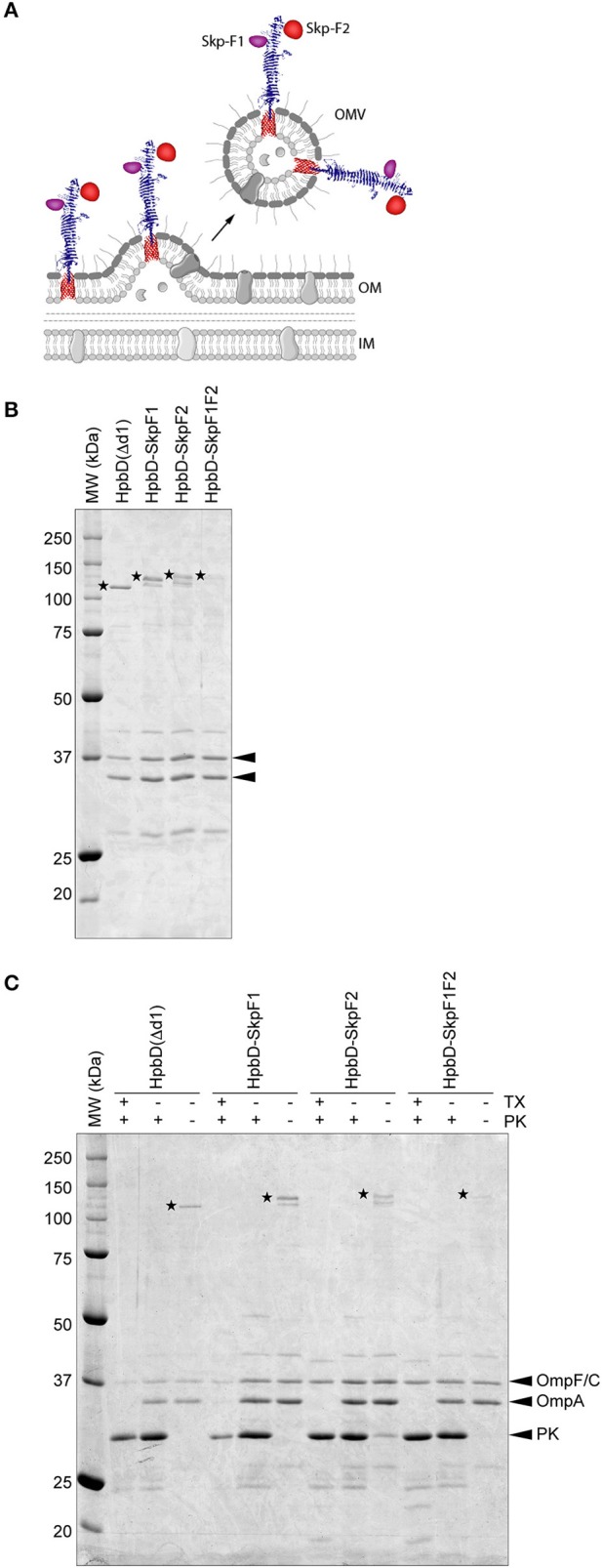
Design and purification of OMV-based Skp vaccine **(A)** Schematic. This figure was created using Servier Medical Art (https://smart.servier.com/) **(B)**. Coomassie stained SDS-PAGE of the OMV based vaccine stocks used in the study. An amount of 0.5 OD units of OMV material was loaded in each lane. HbpD(Δd1) (110 kDa), HbpD-SkpF1 (129 kDa), HbpD-SkpF2 (131 kDa), and HbpD-SkpF1F2 (131 kDa) are marked by asterisks. The OMV porins (OmpF/C and OmpA) are indicated with arrowheads **(C)**. Coomassie stained SDS-PAGE, showing equal amounts of intact (−tx) and Triton X-100 permeabilized (+tx) OMVs treated with (+pk) or without (−pk) Proteinase K. All Hbp-antigens [as well as HbpD(Δd1) control] were exposed at the OMV surface, based on their sensitivity to externally added Proteinase K.

To enable efficient expression of Skp via the Hbp display system, the protein was split into 2 fragments; F1 and F2 (Figure 1A). The fragments were designed to partially overlap, to keep all possible immune epitopes intact. The fragments were either placed individually on Hbp, whereby domain 1 of Hbp (Jong et al., 2012) was replaced (HbpD-SkpF1 and HbpD-SkpF2), or a combination of both fragments fused to a single Hbp, occupying both domain 1 and domain 2 was used (HbpD-SkpF1F2). The three constructs were expressed under control of a lacUV5 promoter in a previously described Δ*tolRA* Δ*msbB* derivative of the attenuated *S. Typhimurium* strain SL3261 (Kuipers et al., 2017). As a negative control, the carrier Hbp that lacks domain 1, HbpD(Δd1), was expressed in the same strain.

This strain produces large amounts of OMVs due to the *tolRA* deletion, and by inactivating the *msbB* gene, penta-acylated LPS is formed, which is less immune-reactive in humans compared to wild-type hexa-acylated lipid A, due to reduced responses via TLR4 activation (Kong et al., 2011). OMVs were isolated from cell-free culture supernatants by ultracentrifugation, after which they were washed with PBS containing a high concentration of NaCl to remove peripherally attached soluble contaminants. OMVs were confirmed to contain the Hbp-antigen chimera by SDS-PAGE (Figure 1B). Importantly, all Hbp-antigen fusions (as well as the HbpD(Δd1) control) were exposed at the OMV surface, based on their sensitivity to externally added Proteinase K (Figure 1C). The C-terminal periplasmic domain of OmpA, which is protease sensitive, was only degraded after permeabilization of the OMVs with Triton X-100, confirming the integrity of the OMVs. Differences in expression levels of the different HbpD-antigen fusion proteins are most likely caused by the difference in complexity of the inserted fragments. However, all Hbp-antigen fusions were clearly visible upon Coomassie staining, indicating that substantial amounts of protein are present.

Mice were immunized three times at 2-week intervals with OMVs expressing the different Hbp-Skp fusions or with Skp-GST. Skp-GST immunizations also included CT as an adjuvant. Immunizations with OMVs lacked exogenous adjuvant. Mice were then inoculated intranasally with ETEC H10407 and evaluated for clinical signs of disease over a 7-day period.

**Figure 2:**
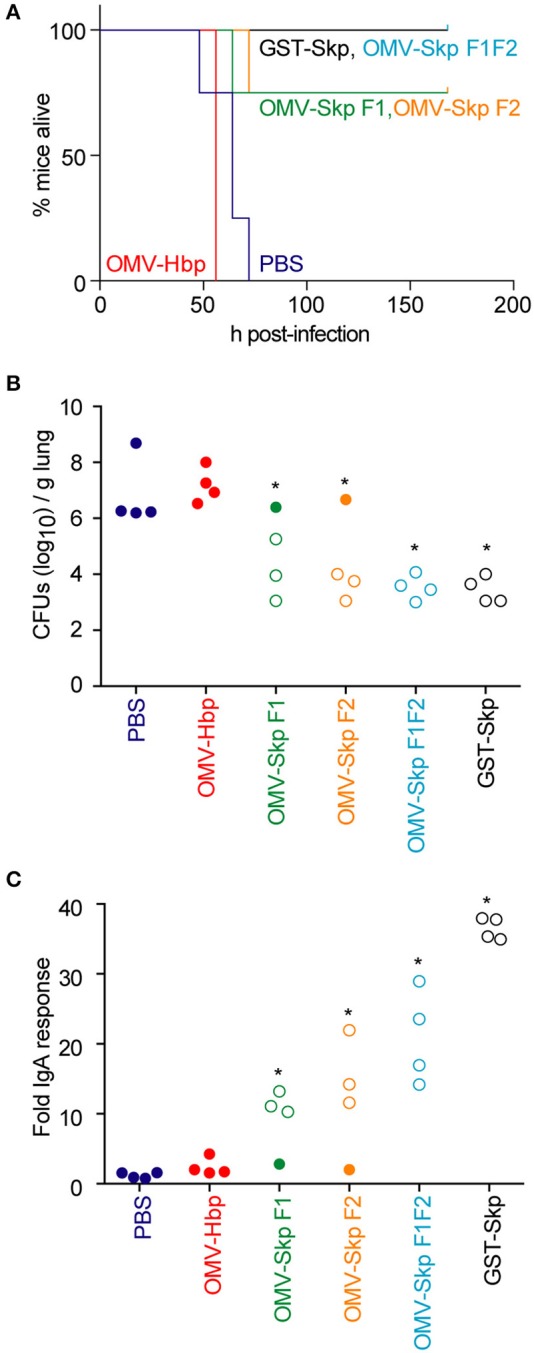
Impact of vaccination on mouse survival after pulmonary challenge with ETEC **(A)**. Mouse survival is plotted as a function of time (h) after mice were inoculated with ETEC H10407 following intranasal immunization with the indicated antigens, *n* = 4. Asterisks indicate significantly different (*p* < 0.05) mouse survival, log-rank test **(B)**. ETEC loads (CFUs/g lung) in mice infected with ETEC H10407 at time of euthanasia or at the end of the study (7 days). Open symbols indicate mice that survived for the duration of the study. Closed symbols indicate mice that were euthanized due to their display of clinical signs of illness, *n* = 4. Asterisks indicate significantly different (*p* < 0.05) ETEC loads in mouse lungs, Kruskal-Wallis test **(C)**. Fold change in mouse fecal IgA concentrations after immunization with the indicated antigens. Open symbols indicate mice that survived for the duration of the study. Closed symbols indicate mice that were euthanized due to their display of clinical signs of illness. *n* = 4. Asterisks indicate significantly different (*p* < 0.05) fecal IgA concentrations, Kruskal-Wallis test.

All control mice [PBS or OMVs containing only the carrier HbpD(Δd1)] died within 48–72 h after challenge, whereas mice immunized with GST-Skp or OMVs containing Skp F1F2 all survived the infection (Figure 2A). OMVs containing either Skp F1 or Skp F2 protected 75 % of mice (Figure 2A).

ETEC concentrations in the lungs of infected mice were inversely correlated with survival (Figure 2B). Whereas, the lungs of control mice contained 10^6^-10^9^ CFUs ETEC at necropsy, mice immunized with OMV-Skp fusions contained only 10^3^-10^6^ CFUs ETEC, similar to results obtained using recombinant GST-Skp.

The concentration of sIgA against Skp in the feces of challenged mice was positively correlated with survival (Figure 2C). Whereas, only baseline IgA concentrations were quantifiable in control mice, mice immunized with OMV-Skp fusions produced robust levels of sIgA. Although the IgA levels were lower as compared to recombinant GST-Skp, the amounts were clearly sufficient for protection.

Collectively, these data demonstrate the utility of using OMVs for the expression and display of antigens for use in vaccine development and validate previously published work (Kumar et al., 2015; Hays et al., 2016) demonstrating that immunization with Skp is efficacious in protecting mice against ETEC challenge. Such OMV formulations may have utility if incorporated into vaccine formulations designed to reduce the global health burden of enteric bacterial pathogens. Since more domains of Hbp are available for antigen replacement, a multivalent vaccine lays within the possibilities of this platform. The exogenous adjuvant-free single formulation can be produced quickly and inexpensively, which make OMVs an attractive vaccine candidate. Alternatively or additionally, purified antigens can be mixed with recombinant OMVs for a wider protective spectrum as in the recently licensed Bexsero MenB vaccine (Esposito et al., 2014).

## Author contributions

MH, DH, and YY performed the experiments. DH and PH designed the study. DH, JL, and PH analyzed the data and wrote the manuscript.

### Conflict of interest statement

JL is employed by Abera Bioscience AB. The other authors declare that the research was conducted in the absence of any commercial or financial relationships that could be construed as a potential conflict of interest.
